# SGLT2 Inhibitors in Diabetic Patients With Cardiovascular Disease or at High Cardiovascular Risk: A Systematic Review and Meta-Analysis of Randomized Controlled Trials

**DOI:** 10.3389/fcvm.2022.826684

**Published:** 2022-04-26

**Authors:** Zinan Zhao, Pengfei Jin, Yatong Zhang, Xin Hu, Chao Tian, Deping Liu

**Affiliations:** ^1^Department of Pharmacy, National Center of Gerontology, Beijing Hospital, Beijing, China; ^2^Institute of Geriatric Medicine, Chinese Academy of Medical Sciences, Beijing, China; ^3^Department of Pharmacy, National Center for Children's Health, Beijing Children's Hospital, Capital Medical University, Beijing, China; ^4^Department of Cardiology, National Center of Gerontology, Beijing Hospital, Beijing, China

**Keywords:** SGLT2 inhibitor, diabetic patient, cardiovascular disease, cardiovascular risk, meta-analysis

## Abstract

**Objective:**

To investigate the effect of sodium-glucose cotransporter-2 inhibitors (SGLT2i) in patients with diabetes with cardiovascular disease (CVD) or at high cardiovascular risk.

**Design:**

Systematic review and meta-analysis of randomized controlled trials (RCTs).

**Data sources:**

Pubmed, Embase, the Cochrane Library, and ClinicalTrial.gov from their inception to August 28, 2021.

**Review methods:**

Randomized control trials (RCTs) assess the effects of SGLT2i in patients with diabetes with cardiovascular disease or at high cardiovascular risk. Primary outcomes included the composite outcome of cardiovascular death (CV death) and hospitalization for heart failure (HHF), HHF, and renal composite outcomes. Secondary outcomes included major adverse cardiovascular events (MACE), CV death, all-cause mortality, and change from the baseline in HbA1c. Additionally, we assessed the effects of treatment in prespecified subgroups on the combined risk of primary and secondary outcomes. These subgroups were based on history of heart failure (HF), estimated glomerular filtration rate (eGFR) levels, and history of hypertension (HTN). A meta-analysis was carried out by using fixed effect models to calculate hazard ratio (HR) or mean difference (MD) between the SGLT2i administrated groups and the control groups.

**Results:**

Four major studies (*n* = 42,568) were included. Primary outcomes showed that SGLT2i was associated with significantly lower risk of CV death/HHF (HR, 0.90; 95% confidence interval, 0.84 to 0.98; P for heterogeneity = 0.01), HHF (HR, 0.84; 95% CI, 0.73 to 0.98; *p* = 0.02), and renal composite outcomes (HR, 0.83; 95%CI, 0.74 to 0.92; *p* = 0.0007) in patients with diabetes with CVD or at high CV risk. Secondary outcome showed that the use of SGLT2i was associated with significant reduction of the HbA1c level (MD, −0.30; 95% CI, −0.36 to −0.23; *p* < 0.00001). In subgroup analyses, SGLT2i significantly reduced the risk of renal composite outcomes in patients without history of HF (HR, 0.75; 95% CI, 0.62 to 0.91; *p* = 0.003 < 0.025). No statistically significant differences were observed in other secondary outcomes and subgroup analyses.

**Conclusions:**

The SGLT2i showed benefits on CV death/HHF, HHF, renal composite outcomes, and HbA1c reduction in patients with diabetes with CVD or at high CV risk. The benefits of improving renal composite outcomes were observed only in patients with diabetes without HF history.

**Systematic Review Registration:**

PROSPERO CRD42021227400

## Introduction

Sodium-glucose co-transporter-2 (SGLT2) inhibitors induce glycosuria, reduce glucose toxicity, and improve insulin sensitivity and β-cell function. By inhibiting sodium and glucose reabsorption from the proximal tubules, the improvement in insulin resistance and natriuresis improved the cardiovascular (CV) mortality in patients with diabetes mellitus (DM).

Large clinical trials and meta-analyses suggest that SGLT2 inhibitors can improve CV and renal outcomes, and, in particular, they reduce the risk of hospitalization for heart failure (HF) in patients with type 2 diabetes mellitus (T2DM) or had a history of HF (1–5).

Current American and European guidelines recommend SGLT2i as second-line therapy after metformin not only for patients with arteriosclerotic cardiovascular disease (ASCVD) but also in those with HF (6, 7). With the advent of new trials, there is a need to look at the outcomes of the population targeted for treatment with SGLT2i as a whole in a large population who had CVD or at high CV risk.

However, no single trial had the adequate power to test the effect of SGLT2i in diabetic patients with CVD or at high CV risk. Our report included four large-scale trials that meet the criteria and allow further rigorous investigation of this issue. The goal of the present meta-analysis was to combine data from all standard large-scale placebo-controlled trials of SGLT2i (canagliflozin, empagliflozin, dapagliflozin, and ertugliflozin) in the population who had CVD or at high CV risk to gain more reliable and accurate evidence of the efficacy in relevant subgroups.

## Methods

### Search Strategy and Selection Criteria

We conducted a systematic search of the literature in August 2021. The data included Pubmed, Embase, the Cochrane Library, and ClinicalTrial.gov. Our search strategy was tailored to each database (eTable 1 in Supplementary File 1).

Studies were included for analysis if they met these criteria: randomized controlled trials of adult individuals (age ≥ 18), whose HbA1c was 7–10.5%, from diabetic patients with CVD or at high CV risk; trials compared SGLT2i in any doses with placebo.

We excluded studies if they were case reports, case series, or observational studies; if they described duplicate data; if they did not report outcomes of interest or primary data; if it was non-diabetic population; if < 1,000 participants; if the follow-up time < 1 year; if the data presented were insufficient to pool for statistical analysis.

The methods were prespecified in a protocol that was registered with the PROSPERO International Prospective Register of Systematic Reviews (crd.york.ac.uk/PROSPERO/display_record.php?ID=CRD42021227400). Approval by a research ethics committee to conduct this meta-analysis was not required in China.

### Data Extraction and Quality Assessment

The studies were initially selected based on their titles and abstracts by two independent authors (ZZ and CT). In the case of any disagreement or uncertainty, full papers were retrieved and reviewed and discussed with a third author (YZ). For each eligible randomized controlled trial, we extracted the study characteristics (e.g., trial registration number, region, year of publication, first author, arms and treatment regimens, follow-up time, participant number), patient characteristics (e.g., average age, gender), and outcome measures.

The quality of randomized controlled trials was assessed by the Cochrane Collaboration Risk of Bias 2 (RoB 2) tool (8). Each item was judged as “yes”, “no”, or “unclear”. Any signaling question answered “yes” indicated a low risk of bias, while “no” showed a high risk of bias. If the answer was uncertain, the domain was judged as having an uncertain risk of bias. Any discrepancies were resolved by consensus, referring to the original articles and consulting with a third reviewer.

### Statistical Analysis

We used hazard ratio (HR) or mean difference (MD) and their associated 95% confidence intervals to assess outcomes, and considered a *P* < 0.05 to be statistically significant in the primary and secondary outcome analysis. For subgroup analysis, 0.05 divided by the number of subgroups was set to be the adjusted *P*-value.

Clinical heterogeneity across studies was assessed by examining variability in participants, baseline data, interventions, and outcomes. Statistic heterogeneity was quantified using the *I*^2^statistic (9). We applied the following thresholds for the interpretation of the *I*^2^: 0–40% might not be important; 30–60% may represent moderate heterogeneity; 50–90% may represent substantial heterogeneity; 75–100% represents considerable heterogeneity. Both the χ^2^ test and the *I*^2^ statistic will be considered for measuring the heterogeneity of effect measures. We will conduct sensitivity analyses based on study quality, where applicable. Analyses were performed in RevMan 5.4.

Primary outcomes included the composite outcome of cardiovascular death (CV death) and hospitalization for heart failure (HHF), HHF, and renal composite outcomes. Secondary outcomes included major adverse cardiovascular events (MACE), CV death, all-cause mortality, and change from the baseline in HbA1c. Subgroup analyses were conducted according to with or without HF, eGFR (≥ 90 ml/min per 1.73 m^2^*v* 60- < 90 ml/min per 1.73 m^2^ *v* < 60 ml/min/1.73 m^2^), with or without hypertension (HTN).

### Patient and Public Involvement

No patients were involved in the definition of the research question or the outcome measures, and interpretation or writing up of results. Data relating to the impact of the intervention on the participants' quality of life were not extracted. Where possible, the results of this meta-analysis will be disseminated to the patient community or individual patients and families through the investigators of this meta-analysis.

## Results

### Studies Retrieved and Characteristics

Of 2,978 studies eligible for inclusion in the initial screen, 2,041 were excluded for not meeting the inclusion criteria and repetition. After a full-text review, four studies included 15 reported articles that were included in the final analysis (see the PRISMA flow diagram in Supplementary File 2). The definition of CVD or at high CV risk in each included study is in eTable 2, Supplementary File 1.

Table 1 shows the main characteristics of the eligible trials. The risk of bias assessment was performed for each RCT and summarized. All trials had an unclear risk of other bias (see eFigure 1 in Supplementary File 1).

**Table 1:** An overview of the main characteristics of the four trial populations at the baseline.

	**VERTIS CV** **(****10**, **11****)**		**DECLARE-TIMI 58** **(****5**, **12**, **13****)**		**CANVAS Program** **(****14**, **15****)** **(CANVAS, CANVAS-R)**		**EMPA-REG OUTCOME** **(****3**, **16**, **17****)**	
Year	2020		2019		2018		2017	
NCT number	NCT01986881		NCT01730534		NCT01032629, NCT01989754		NCT01131676	
Conditions	T2DM & ASCVD		T2DM & atherosclerotic CVD/atherosclerotic CV risk		T2DM & elevated cardiovascular risk		T2DM & established CVD	
Median follow-up time	6.1 years		5.2 years		188.2 weeks		4.6 years	
Interventions	Ertugliflozin 5/15 mg, once daily	Placebo	Dapagliflozin 10 mg, once daily	Placebo	Canagliflozin 100/300 mg, once daily	Placebo	Empagliflozin 10/25 mg, once daily	Placebo
Participants	5,499	2,747	8,582	8,578	5,795	4,347	4,687	2,333
Age (Mean ± SD)	64.4 ± 8.1	64.4 ± 8.0	63.9 ± 6.8	64.0 ± 6.8	63.2 ± 8.3	63.4 ± 8.2	63.1 ± 8.6	63.2 ± 8.8
Gender				
Male	3,866 (70.3%)	1,903 (69.3%)	5,411 (63.1%)	5,327 (62.1%)	3,759 (64.9%)	2,750 (63.3%)	3,336 (71.2%)	1,680 (72.0%)
Female	1,633 (29.7%)	844 (30.7%)	3,171 (36.9%)	3,251 (37.9%)	2,036 (35.1%)	1,597 (36.7%)	1,351 (28.8%)	653 (28.0%)
DM	100%		100%		100%		100%	
Concomitant treatments				
Other AHA	Insulin, Metformin, Sulfonylurea, and/or Glinide		Insulin, Metformin, and/or Sulfonylurea		Insulin, Metformin, and/or Sulfonylurea		Insulin, Metformin, and/or Sulfonylurea	
CV medications	ACEI/ARB, β-Blocker, and/or MRA		Antiplatelet therapy, ACEI/ARB,β-Blocker, Statin and/or ezetimibe		Antithrombotic, ACEI/ARB, β-Blocker, Statin and/or Diuretic		ACEI/ARB, β-Blocker, CCB, Statin and/or Diuretic	

*Data are n (%) or mean (SD). ACEI, angiotensin-converting enzyme inhibitor; AHA, anti-hyperglycemic agents; ARB, angiotensin receptor blocker; ASCVD, atherosclerotic cardiovascular disease; CV, cardiovascular; CVD, cardiovascular disease; CCB, calcium channel blocker; DM, diabetes mellitus; HF, heart failure; MRA, mineral ocorticoid receptor antagonist; T2DM, type 2 diabetes mellitus*.

### Primary Outcomes

**Figure 1:**
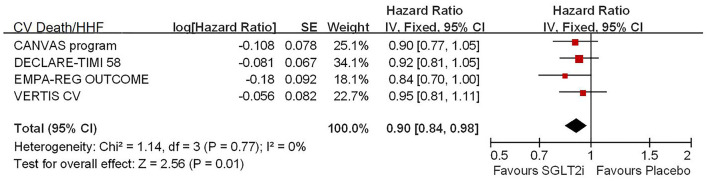
Meta-analysis of SGLT2i on CV death or HHF.

**Figure 2:**
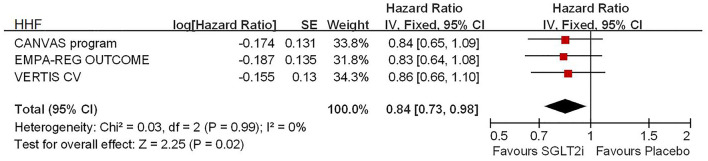
Meta-analysis of SGLT2i on HHF.

**Figure 3:**
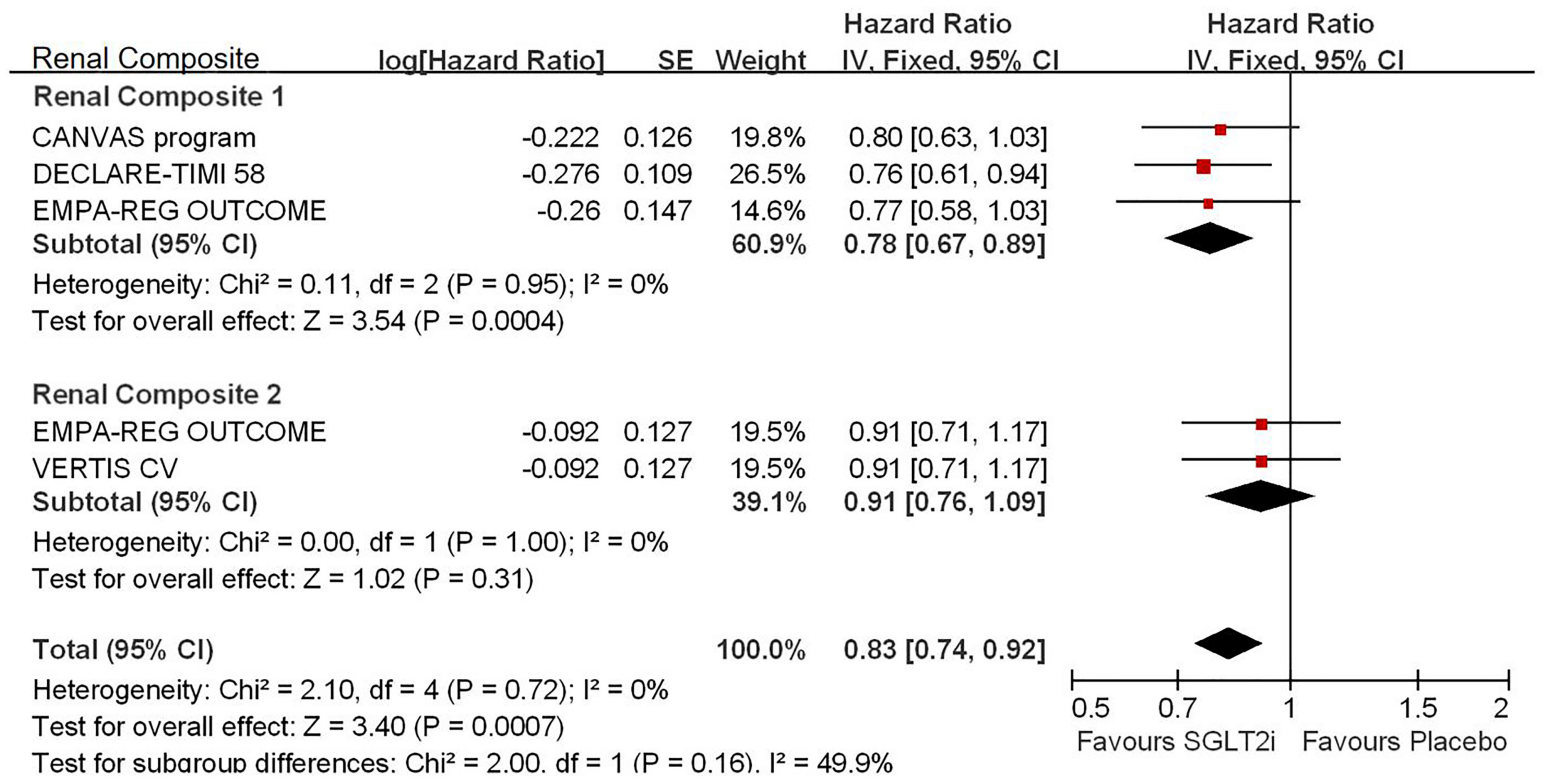
Meta-analysis of SGLT2i on renal composite outcomes. Renal Composite 1 concluded 40% decrease in eGFR, end-stage kidney disease, or renal death; renal Composite 2 included renal death, renal dialysis/transplant, or doubling of serum creatinine from the baseline.

Among 42,568 patients in the four trails, the use of SGLT2i was associated with significantly reduced CV death/HHF (HR, 0.84; 95% CI, 0.73 to 0.98; *p* = 0.02; Figure 1), HHF (HR, 0.84; 95% CI, 0.73 to 0.98; *p* = 0.02; Figure 2), and renal composite outcomes (HR, 0.83; 95% CI, 0.74 to 0.92; *p* = 0.0007; Figure 3; renal Composite 1 concluded 40% decrease in eGFR, end-stage kidney disease, or renal death; renal Composite 2 included renal death, renal dialysis/transplant, or doubling of serum creatinine from the baseline) in diabetic patients with CVD or at high CV risk.

### Secondary Outcomes

The effects of SGLT2i on MACE, CV death, and all-cause mortality were not statistically significant in diabetic patients with CVD or at high CV risk. SGLT2i was associated with significant reduction of the HbA1c level (MD, −0.30; 95% CI, −0.36 to −0.23; *p* < 0.00001) (see eFigures 2–5 in Supplementary File 1).

### Subgroup Analyses

To explore the reasons for heterogeneity, we conducted several subgroup analyses to investigate whether the effects of SGLT2i were affected by the presence or absence of HF, renal function (eGFR), or the presence or absence of HTN.

#### CV Death/HHF

For the history with or without HF, no evidence was found for the treatment-by-subgroup interactions (see eFigure 6 in Supplementary File 1).

#### HHF

For the history with or without HF, no evidence was found for the treatment-by-subgroup interactions (see eFigure 7 in Supplementary File 1).

The SGLT2i was associated with a significantly lower risk of HHF in the total patients with diabetes, but the difference was not observed in the subgroup analyses of different GFR levels (see eFigure 8 in Supplementary File 1).

For the history with HTN or without HTN, no significant differences were found in SGLT2i in diabetic patients with CVD or at high CV risk (see eFigure 9 in Supplementary File 1).

#### Renal Composite Outcomes

**Figure 4:**
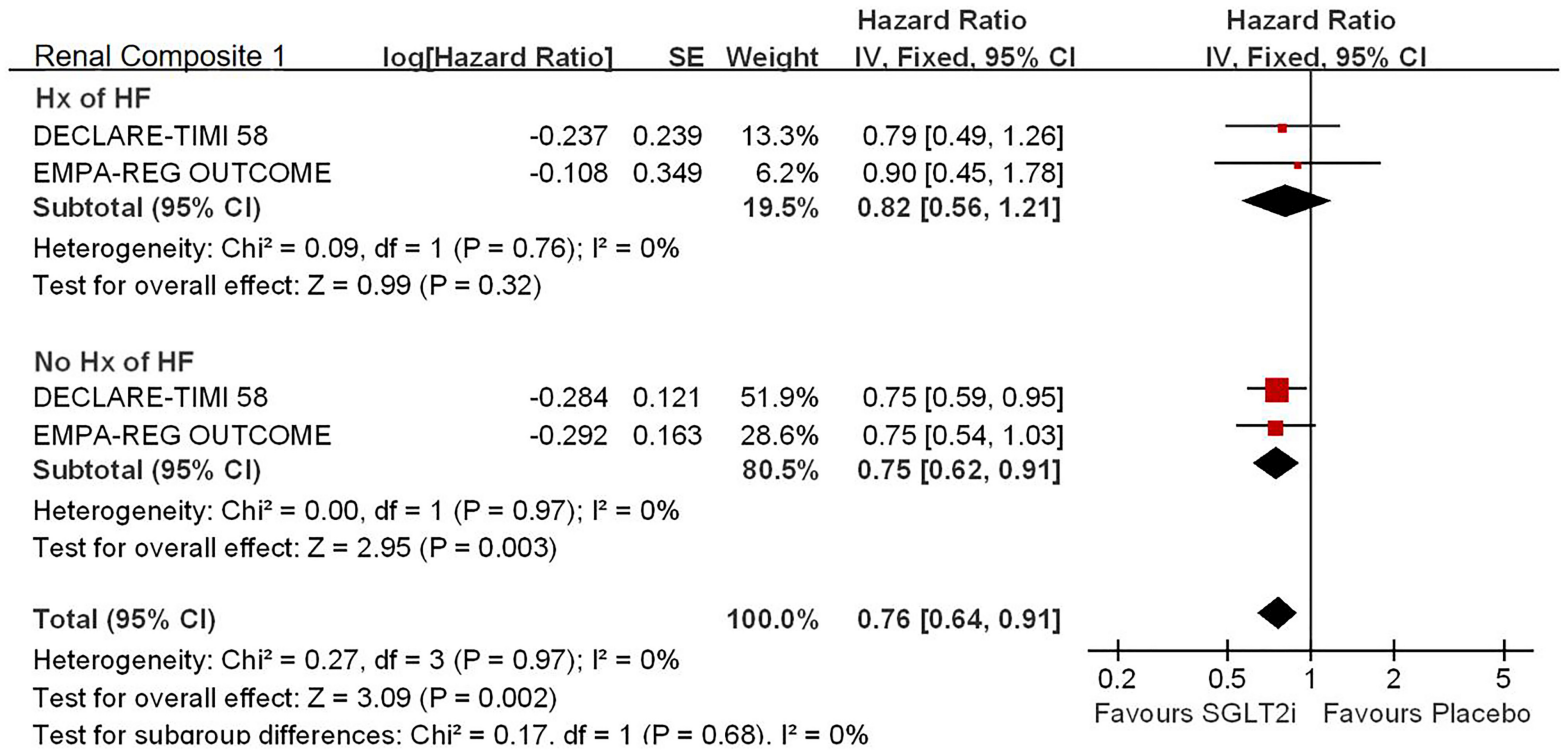
Subgroup analysis of meta-analysis on HHF by eGFR levels.

For the history with or without HF, SGLT2i significantly reduced the risk of renal composite outcomes in patients without history of HF (HR, 0.75; 95% CI, 0.62 to 0.91; *p* = 0.003 < 0.025; *see* Figure 4).

The SGLT2i was associated with a significantly lower risk of renal composite outcomes in the total patients with diabetes but the difference was not observed in the subgroup analyses of different eGFR levels (see eFigure 10 in Supplementary File 1).

#### MACE

For the differences of eGFR levels, we found no evidence for the treatment-by-subgroup interactions (see eFigure 11 in Supplementary File 1).

#### CV Death

For the presence or absence of HF, no significant differences were found for the treatment-by-subgroup interactions (see eFigure 12 in Supplementary File 1).

#### All-Cause Mortality

For the presence or absence of HF, no significant differences were found for treatment-by-subgroup interactions in different age groups (see eFigure 13 in Supplementary File 1).

## Discussion

### Principal Findings

Our report is the first meta-analysis of the four major RCTs with a total of 42,568 participants, assessing the effects of SGLT2i in diabetic patients with CVD or at high CV risk, including established CVD, elevated CV risk, atherosclerotic cardiovascular disease (ASCVD), and atherosclerotic CV risk. Our report of SGLT2i trials expands on previous meta-analysis (12); our data make several patterns clear.

In diabetic patients with CVD or at high CV risk, first, SGLT2i led to a benefit on CV death/HHF, HHF, and renal composite outcomes. Second, for particular outcomes, the clinical effects of SGLT2i depend on the patient population in which they are used. The significant reduction in renal composite outcomes was observed only in patients without HF history.

### Comparison With Previous Studies

The beneficial role of SGLT2 inhibitors in patients with diabetes with CVD or at high CV risk was not only through a directly hypoglycemic action, but also in multitude pleiotropic actions through the CV mechanisms. Studies showed that aspects, such as the pre-load reduction and the cardiac energetics improvement, through an increase in ketones' supply, should be involved in the SGLT2 inhibitors positive effects on CV and renal outcomes (18, 19).

Before our report, there were two major meta-analyses (1, 2) that estimated the effects of SGLT2i on the composite CV outcomes in patients with HFrEF and established ASCVD. The benefits on composite CV outcomes for HF or established ASCVD were of similar magnitude regardless of the presence of established CVD or history of HF. Our meta-analysis divided the endpoints into different populations, including patients with or without history of patients with HF in different renal function (eGFR), and patients with or without a history of HTN.

Heart failure is a complex and multifaceted disease that leads to multisystemic mechanisms. SGLT2 inhibitors are a very valid tool for HF with reduced ejection fraction (HFrEF) (20). It is our believe that, due to the current outcomes which did not distinguish the HFrEF and HF with preserved ejection fraction (HFpEF) with the population included, SGLT2 inhibitors may be a possible important role for HFpEF.

However, all the results reported were regarding a wide spectrum of disease (including HF and renal impairment) manifestations and stages. Recent studies have indicated that it is important to define the best administration timing and the most suitable patients to maximize the SGLT2 inhibitors-derived beneficial effects (20). Those may provide new perspectives for the management of patients with diabetes along with HF or renal impairment.

The SGLT2i induces osmotic diuresis and can thereby affect CV activity in patients with hyperglycemia (21). However, the exact mechanisms of the salutary effects of SGLT2i to CV system remain unclear (22). Our results support new recommendations that suggest SGLT2i be used in patients who had CVD or at high CV risk on multiple outcomes, such as CV death/HHF, HHF, and renal composite outcomes.

## Limitations

Several limitations should be acknowledged. First, the exact inclusion criteria (Supplementary File 1) and definitions of endpoints varied among the included trials, but only slightly. Second, limited by the availability of data, the definition of HF was not specified. Classification of HF (preserved or reduced HF) may influence the final outcome. Third, due to the lack of racial data, the analysis of CV risk factor of ethnicity may not be performed. Fourth, our study mainly focused on the CV outcomes, but did not present the adverse outcomes such as urinary tract infections, female genital mycotic infections, and dyslipidemia. Sixth, there may be a possible interference in the outcomes by concomitant therapeutic treatments in terms of pharmacokinetic and/or pharmacodynamic interaction. Further research into this issue is expected.

## Conclusions

Our meta-analysis establishes a moderate evidence base, confirming the important role of SGLT2i in reducing CV death/HHF, HHF, and renal composite outcomes in diabetic patients who had CVD or at high CV risk. Furthermore, subgroup analyses confirmed accurate benefits of SGLT2i on renal composite outcomes, which had no history of HF.

## Strengths and Limitations of This Study

- This is the first meta-analysis of the four major RCTs with a total of 42,568 participants assessing the effects of SGLT2i in diabetic patients with CVD or at high CV risk, including established CVD, elevated CV risk, ASCVD, and atherosclerotic CV risk.
- In patients with diabetes with CVD or at high CV risk, first, SGLT2i led to a moderate benefit on CV death/HHF, HHF, and renal composite outcomes.
- The benefits of improving renal composite outcomes were observed only in patients with diabetes without HF history.
- No benefits were observed in MACE, CV death and all-cause mortality, and other subgroup analyses.
- The exact inclusion criteria (Supplementary File 1) and definitions of endpoints varied among the included trials, but only slightly.

## Data Availability Statement

The original contributions presented in the study are included in the article/Supplementary Material, further inquiries can be directed to the corresponding author.

## Author Contributions

ZZ contributed to study design, data extraction, quality assessment, statistical analysis, data interpretation, and drafting of the manuscript. YZ and CT contributed to data extraction, quality assessment, and critical review of the manuscript. PJ and XH contributed to data interpretation and critical review of the manuscript. DL is the guarantor of this work and, as such, had full access to all the data in the study and takes responsibility for the integrity of the data and the accuracy of the data analysis. All authors contributed to the article and approved the submitted version.

## Funding

This work was financially supported by China National Key Research and Development Program (Grant No.: 2020YFC2003001); China National Key Research and Development Program (Grant No.: 2020YFC2009000); and China National Key Research and Development Program (Grant No.: 2020YFC2008300).

## Conflict of Interest

The authors declare that the research was conducted in the absence of any commercial or financial relationships that could be construed as a potential conflict of interest.

## Publisher's Note

All claims expressed in this article are solely those of the authors and do not necessarily represent those of their affiliated organizations, or those of the publisher, the editors and the reviewers. Any product that may be evaluated in this article, or claim that may be made by its manufacturer, is not guaranteed or endorsed by the publisher.
