# Supplementary material for: SGLT2 Inhibitors in Diabetic Patients With Cardiovascular Disease or at High Cardiovascular Risk: A Systematic Review and Meta-Analysis of Randomized Controlled Trials
Source: Front Cardiovasc Med. 2022 Apr 26;9:826684. doi: 10.3389/fcvm.2022.826684 (PMC9087280; doi:10.3389/fcvm.2022.826684)

Supplementary file 1

# *eTable 1*. Literature search strategy.

**Pubmed**

| Searches | | Results |
| --- | --- | --- |
| ^#^1 | (((((((((((((((((sodium glucose cotransporter[Text Word]) OR (sodium-glucose-cotransporter-2 inhibitors[Text Word])) OR (sglt-2[Text Word])) OR (sglt2[Text Word])) OR (canagliflozin[Text Word])) OR (invokana[Text Word])) OR (dapagliflozin[Text Word])) OR (farxiga[Text Word])) OR (empagliflozin[Text Word])) OR (jardiance[Text Word])) OR (ertugliflozin[Text Word])) OR (luseogliflozin[Text Word])) OR (tofogliflozin[Text Word])) OR (ipragliflozin[Text Word])) OR (sergliflozin[Text Word])) OR (sotagliflozin[Text Word])) OR (remo gliflozins[Text Word])) OR (bexagliflozin[Text Word]) | 7,542 |
| ^#^2 | (((((randomized controlled trial[Publication Type]) OR (controlled clinical trial[Publication Type])) OR (randomized[Text Word])) OR (clinical trial[Text Word])) OR (trial[Title])) OR (placebo[Text Word]) | 1,409,786 |
| ^#^3 | (((((((((((((((("cardiovascular disease"[Text Word]) OR ("cardiocerebrovascular disease"[Text Word])) OR ("coronary artery disease"[Text Word])) OR ("coronary heart disease"[Text Word])) OR ("ischemic heart disease"[Text Word])) OR ("myocardial infarction"[Text Word])) OR ("cardiovascular events"[Text Word])) OR ("cardiac events"[Text Word])) OR ("cerebrovascular disease"[Text Word])) OR (stroke[Text Word])) OR ("cerebral infarction"[Text Word])) OR (mortality[Text Word])) OR (fatal[Text Word])) OR (death[Text Word])) OR ("MACE"[Text Word])) OR ("cardiovascular death"[Text Word])) OR ("heart failure"[Text Word]) | 2,750,131 |
| ^#^4 | ^#^1 AND ^#^2 AND ^#^3 | 939 |

**Embase**

| Searches | | Results |
| --- | --- | --- |
| ^#^1 | 'sodium glucose cotransporter'/exp OR 'sodium-glucose-cotransporter-2 inhibitors' OR 'sglt-2' OR 'sglt2' OR 'canagliflozin'/exp OR 'invokana'/exp OR 'dapagliflozin'/exp OR 'farxiga'/exp OR 'empagliflozin'/exp OR 'jardiance'/exp OR 'ertugliflozin'/exp OR 'luseogliflozin'/exp OR 'tofogliflozin'/exp OR 'ipragliflozin'/exp OR 'sergliflozin'/exp OR 'sotagliflozin'/exp OR 'remo gliflozins' OR 'bexagliflozin' | 12,132 |
| ^#^2 | 'randomized controlled trial' OR 'randomized controlled trial'/exp | 840,978 |
| ^#^3 | 'cardiovascular disease'/exp OR 'cardiocerebrovascular disease' OR 'coronary artery disease'/exp OR 'coronary heart disease'/exp OR 'ischemic heart disease'/exp OR 'myocardial infarction'/exp OR 'cardiovascular events' OR 'cardiac events' OR 'cerebrovascular disease'/exp OR 'stroke'/exp OR 'cerebral infarction'/exp OR 'mortality'/exp OR 'fatal' OR 'death'/exp OR 'mace'/exp OR 'cardiovascular death'/exp OR 'heart failure'/exp | 5,703,849 |
| ^#^4 | ^#^1 AND ^#^2 AND ^#^3 | 596 |

**the Cochrane Library**

| Searches | | Results |
| --- | --- | --- |
| ^#^1 | (sodium glucose cotransporter) OR (sodium-glucose-cotransporter-2 inhibitors) OR (sglt-2) OR (sglt2) OR (canagliflozin) | 1,897 |
| ^#^2 | (invokana) OR (dapagliflozin) OR (farxiga) OR (empagliflozin) OR (jardiance) | 2,067 |
| ^#^3 | (ertugliflozin) OR (luseogliflozin) OR (tofogliflozin) OR (ipragliflozin) OR (sergliflozin) | 471 |
| ^#^4 | (sotagliflozin) OR (remo gliflozins) OR (bexagliflozin) | 124 |
| ^#^5 | ^#^1 OR ^#^2 OR ^#^3 OR ^#^4 | 3,341 |
| ^#^6 | (randomized controlled trial):pt OR (controlled clinical trial):pt OR (randomized):ab | 1,329,971 |
| ^#^7 | (trial):ti,ab,kw OR (placebo) | 966,761 |
| ^#^8 | ^#^6 OR ^#^7 | 1,380,021 |
| ^#^9 | (cardiovascular disease) OR (cardiocerebrovascular disease) OR (coronary artery disease) OR (coronary heart disease) OR (ischemic heart disease) | 69,412 |
| ^#^10 | (myocardial infarction) OR (cardiovascular events) OR (cardiac events) OR (cerebrovascular disease) OR (stroke) | 113,927 |
| ^#^11 | (cerebral infarction) OR (mortality)) OR (fatal) OR (death) OR (MACE) | 144,761 |
| ^#^12 | (cardiovascular death) OR (heart failure) | 49,614 |
| ^#^13 | ^#^9 OR ^#^10 OR ^#^11 OR ^#^12 | 266,531 |
| ^#^14 | ^#^5 AND ^#^8 AND ^#^13 | 975 |

**ClinicalTrial.gov**

Basic search

243 Studies found for: SGLT2 inhibitor | Completed Studies | Studies With Results | Interventional Studies. Also searched for Sodium-Glucose Transporter 2 Inhibitors and SODIUM-GLUCOSE TRANSPORTER 2.

# *eTable 2*. Definition of cardiovascular disease and cardiovascular risk in the included trials.

| **Trial** | **Definition of cardiovascular disease** | **Definition of cardiovascular risk** |
| --- | --- | --- |
| **VERTIS CV** | Atherosclerotic cardiovascular disease (ASCVD) including coronary, cerebrovascular and peripheral vascular disease. | Not included. |
| **DECLARE-TIMI 58** | Atherosclerotic cardiovascular disease (defined as clinically evident ischemic heart disease, ischemic cerebrovascular disease, or peripheral artery disease). | Multiple risk factors for atherosclerotic cardiovascular disease (men 55 years of age or older or women 60 years of age or older who had one or more traditional risk factors, including hypertension, dyslipidemia (defined as a low-density lipoprotein cholesterol level >130 mg per deciliter [3.36 mmol per liter] or the use of lipid-lowering therapies), or use of tobacco). |
| **CANVAS Program**  **(CANVAS, CANVAS-R)** | ≥30 years of age with a history of symptomatic atherosclerotic cardiovascular events defined as stroke, MI, hospitalization for unstable angina, coronary artery bypass grafting, percutaneous coronary intervention, peripheral revascularization (surgical or percutaneous), and symptomatic with documented hemodynamically significant carotid or peripheral vascular disease or amputation secondary to vascular disease (secondary prevention cohort). | ≥50 years of age with no prior cardiovascular events but with ≥2 of the following cardiovascular risk factors: duration of diabetes mellitus ≥10 years, systolic blood pressure >140 mmHg on ≥1 antihypertensive agents, current smoker, microalbuminuria or macroalbuminuria, or high-density lipoprotein cholesterol <1 mmol/L (primary prevention cohort). |
| **EMPA-REG OUTCOME** | Ischemic Heart Disease: MI (>2 months prior to informed consent), or Multivessel CAD (50% stenosis in ≥2 major coronary arteries or the left main artery (i.e., previous revascularization ≥2 major coronary arteries or left main artery; or combination of revascularization in at least 1 main artery and 50% stenosis in 1 main coronary artery), OR Single vessel CAD (50% stenosis in ≥1 main coronary artery and a positive stress test OR hospital discharge for unstable angina ≤12 months prior to consent), or Unstable angina with evidence of single or multivessel CAD (>2 months prior to consent), OR History of stroke (ischemic or hemorrhagic), OR Peripheral artery disease prior revascularization, OR previous limb or foot amputation due to circulatory insufficiency; or angiographic evidence of significant (>50%) peripheral artery stenosis in at least one limb; or evidence from a non-invasive measurement of significant (>50% or as reported as hemodynamically significant) peripheral artery stenosis in at least one limb; or ankle brachial index of < 0.9 in at least one limb. | Not included. |

# *eFigure 1*. Risk of bias summary (assessed by the Cochrane Collaboration Risk of Bias 2 tool): review authors' judgements about each risk of bias item for each included study.


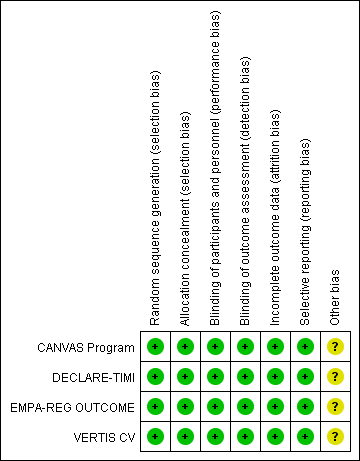


Note: Risk of bias levels; low (green or “+”), Unclear (yellow or “?”), High (red or “-”).

# *eFigure 2.* Meta-analysis of SGLT2i on MACE.


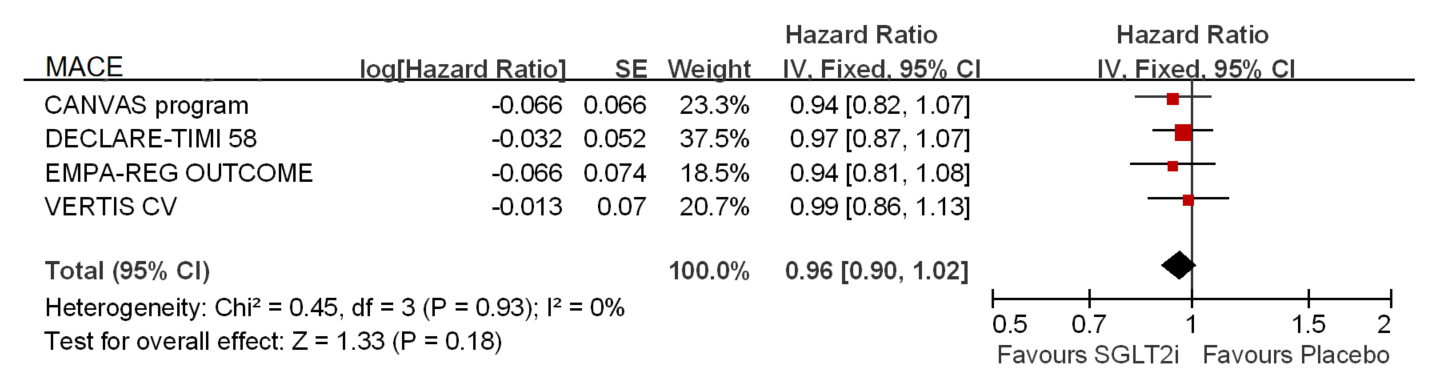


# *
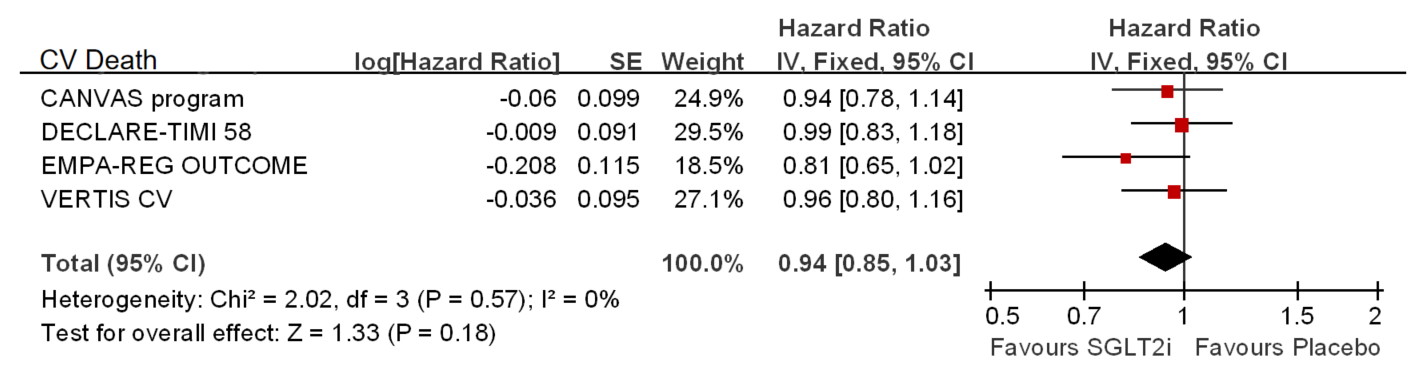
eFigure 3*. Meta-analysis of SGLT2i on CV Death.

# **
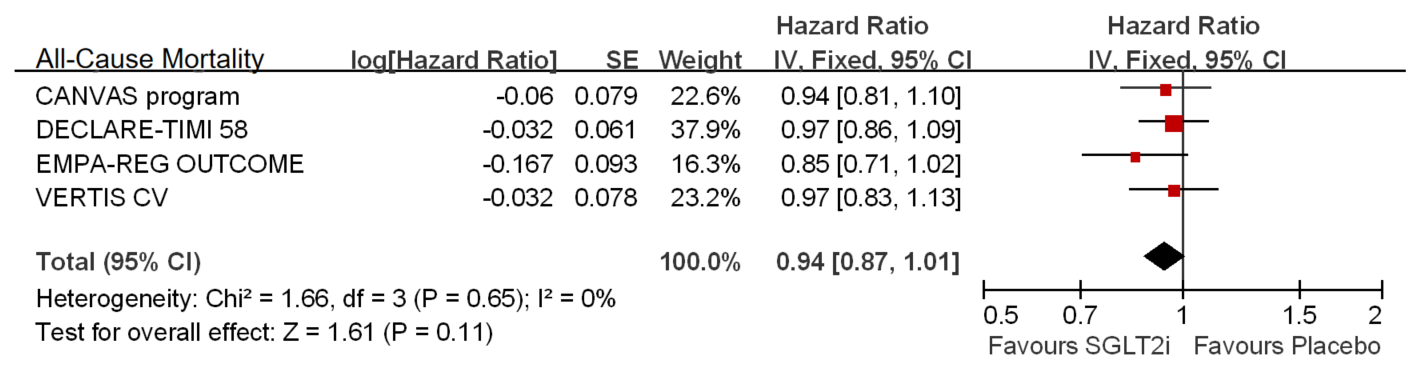
*eFigure 4.* Meta-analysis of SGLT2i on All-Cause Mortality.**

***efigure 5***. **Meta-analysis of SGLT2i on change from baseline in HbA1c.**


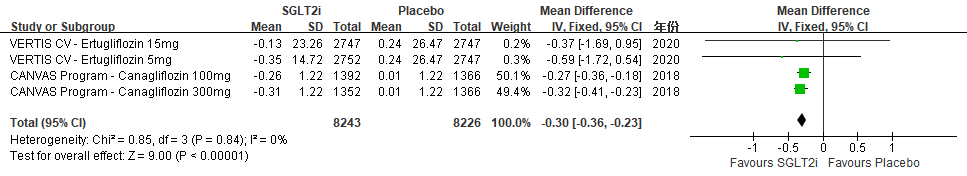


***eFigure 6*. Subgroup analysis of Meta-analysis on CV Death/HHF by the history (Hx) of HF.**


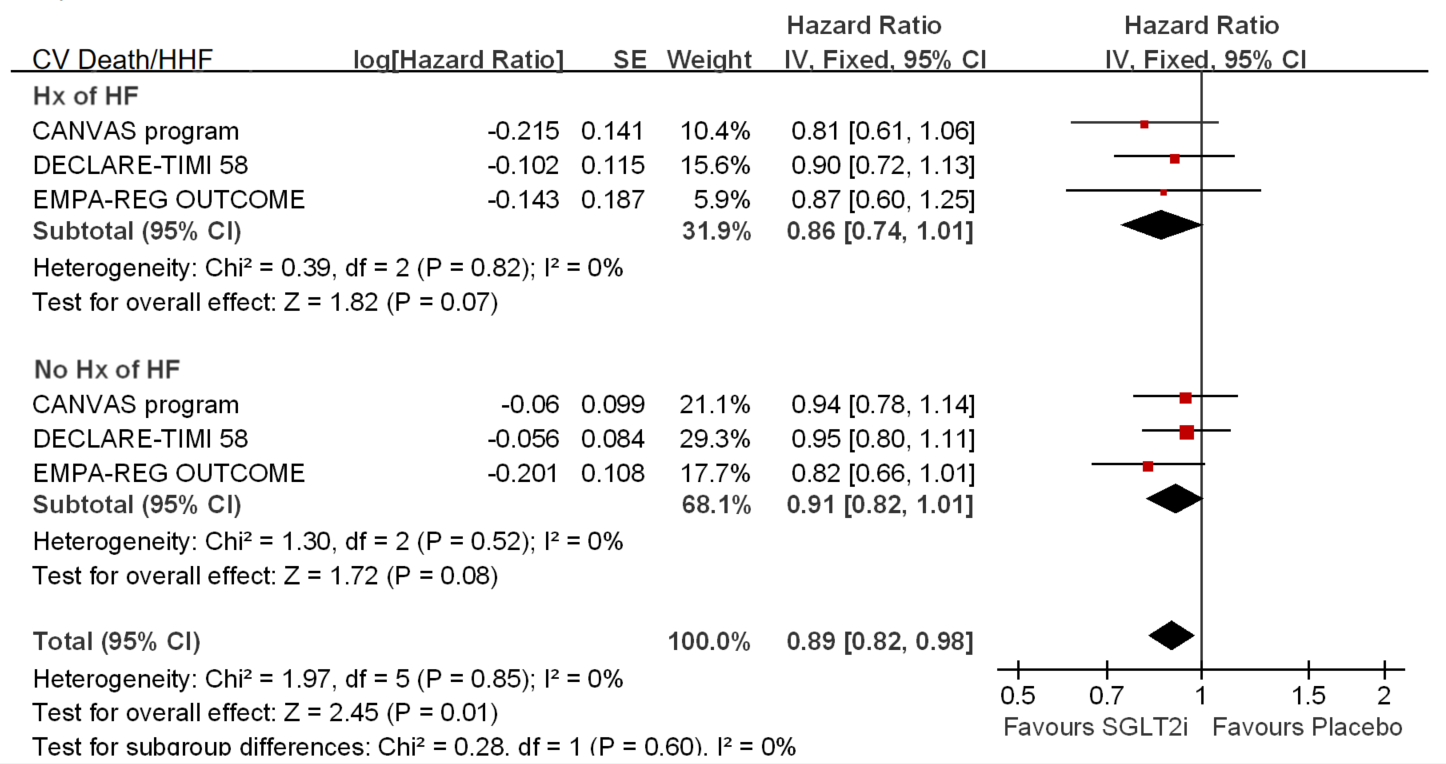


***eFigure 7*. Subgroup analysis of Meta-analysis on HHF by the hx of HF.**


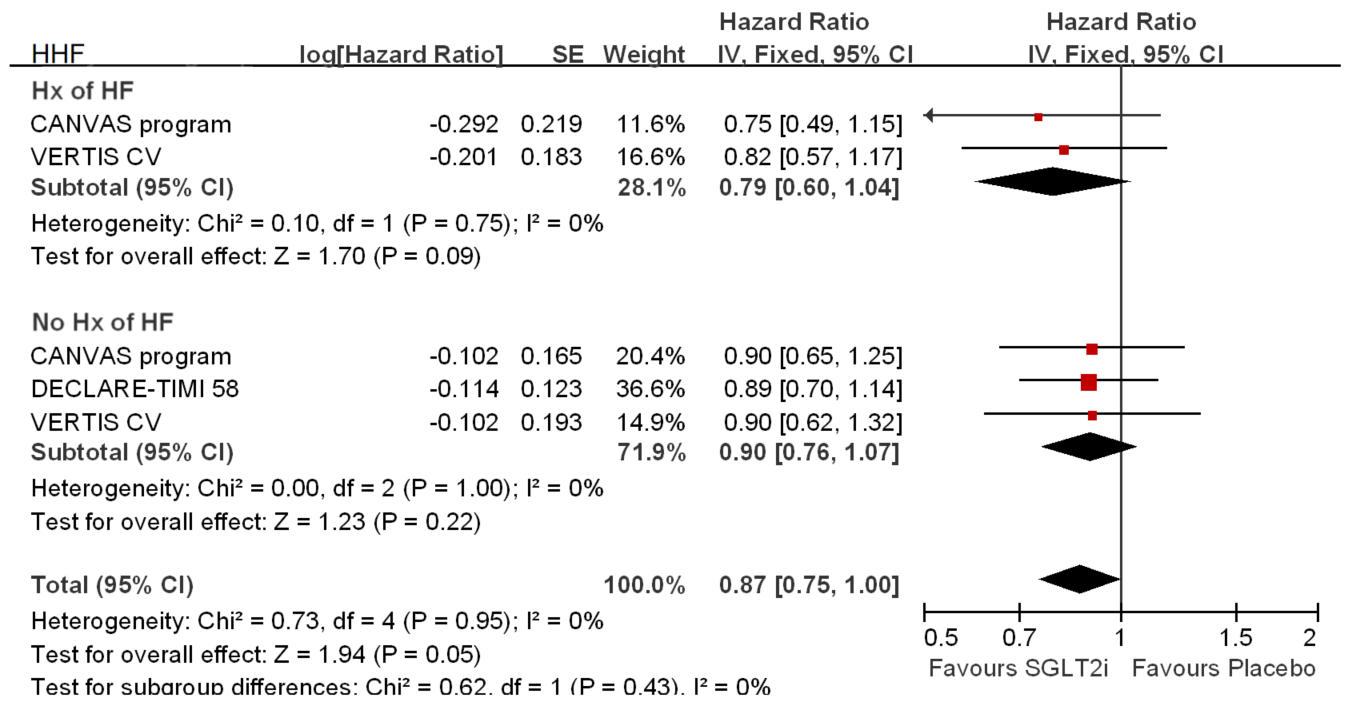


***eFigure 8.* Subgroup analysis of Meta-analysis on HHF by the eGFR levels.**

***
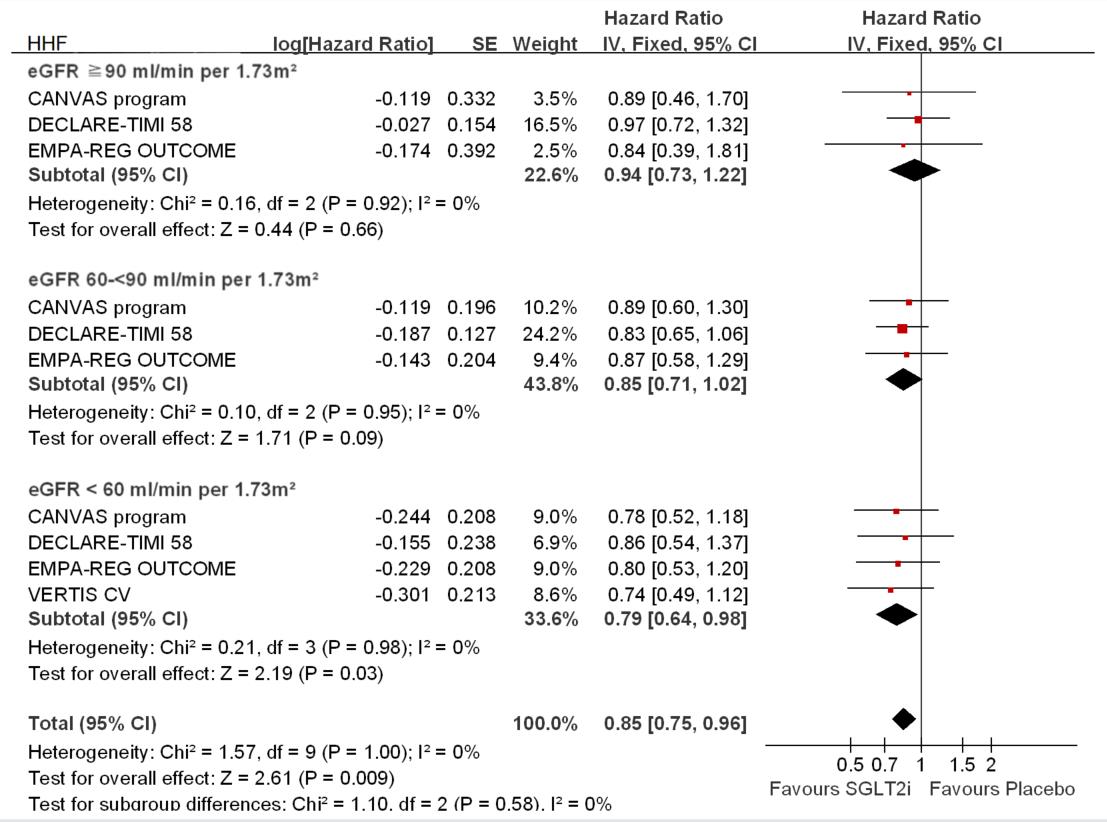
***

***eFigure 9.* Subgroup analysis of Meta-analysis on HHF by the hx of HTN.**


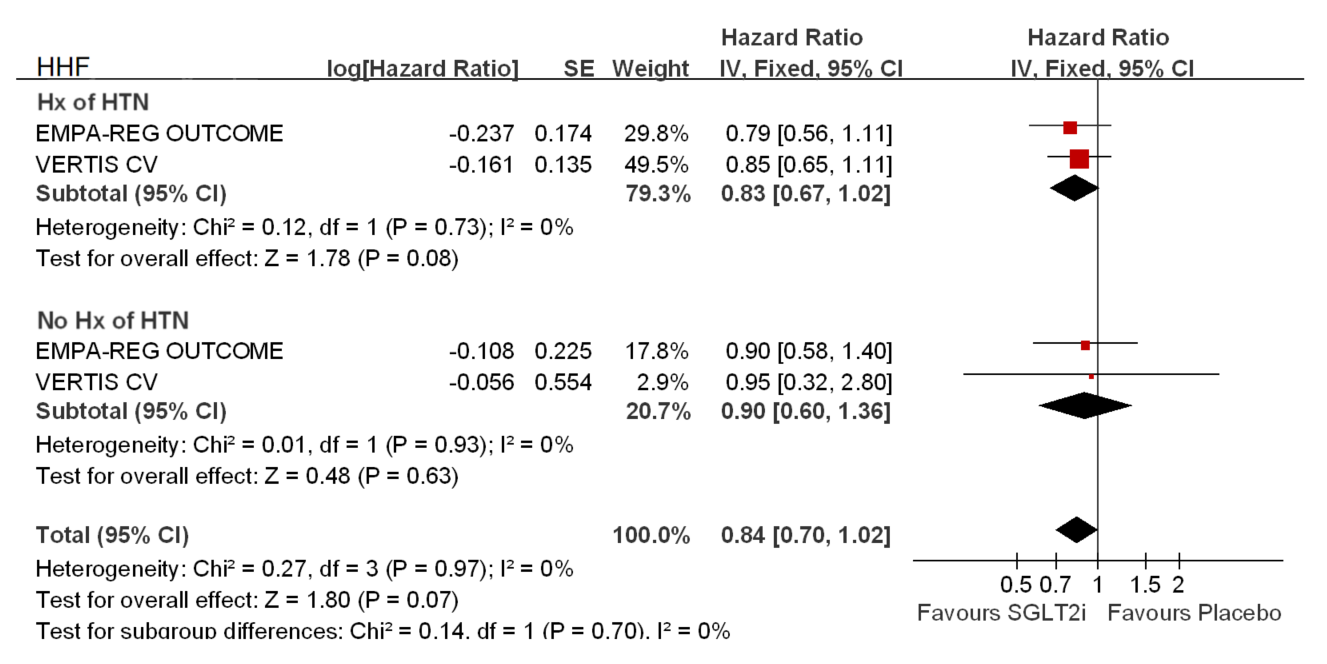


***eFigure 10.* Subgroup analysis of Meta-analysis on renal composite outcomes by the eGFR levels.**


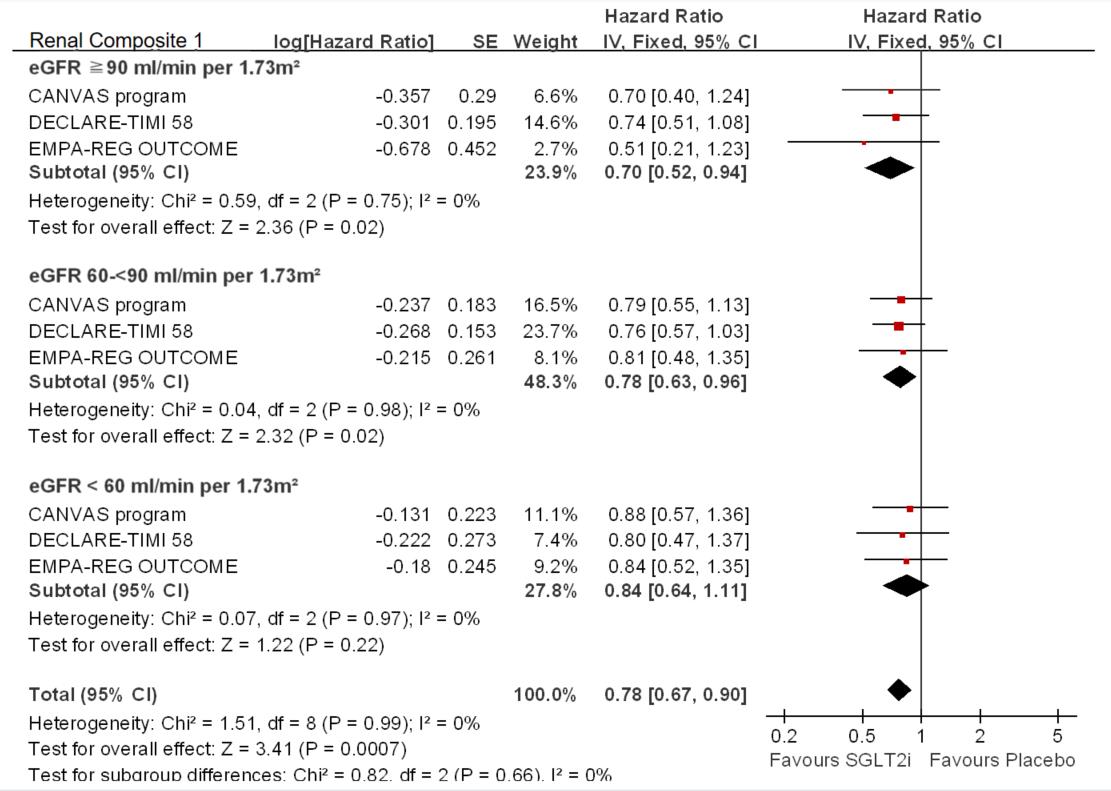


***eFigure 11.* Subgroup analysis of Meta-analysis on MACE by the eGFR levels.**


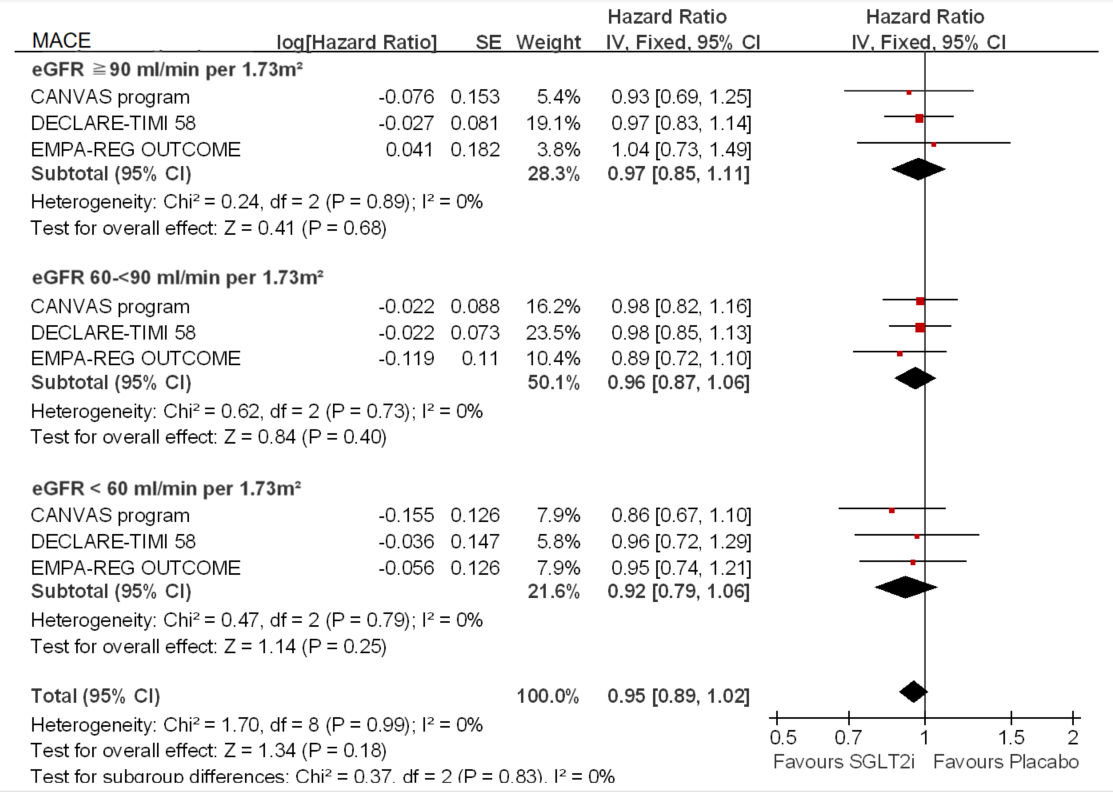


***eFigure 12.* Subgroup analysis of Meta-analysis on CV Death by the hx of HF.**


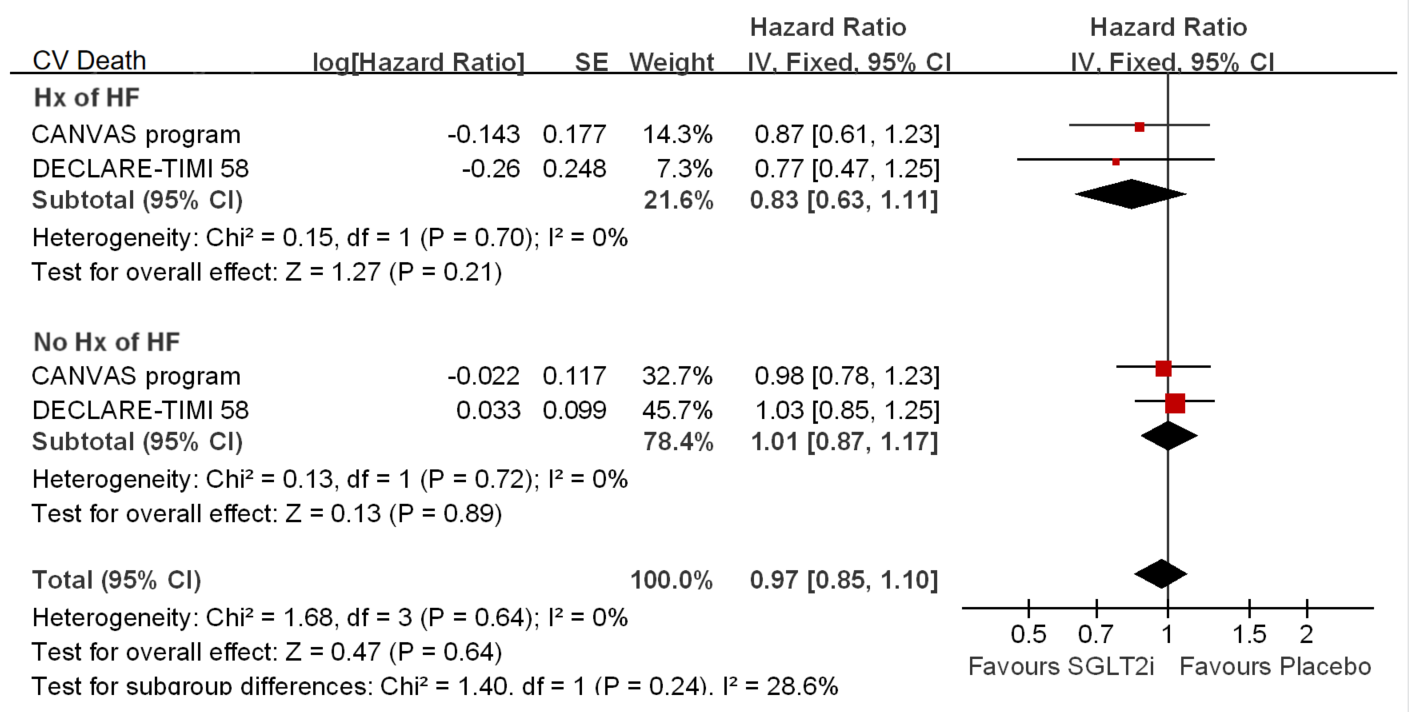


***eFigure 13.* Subgroup analysis of Meta-analysis on All-Cause Mortality by the hx of HF.**


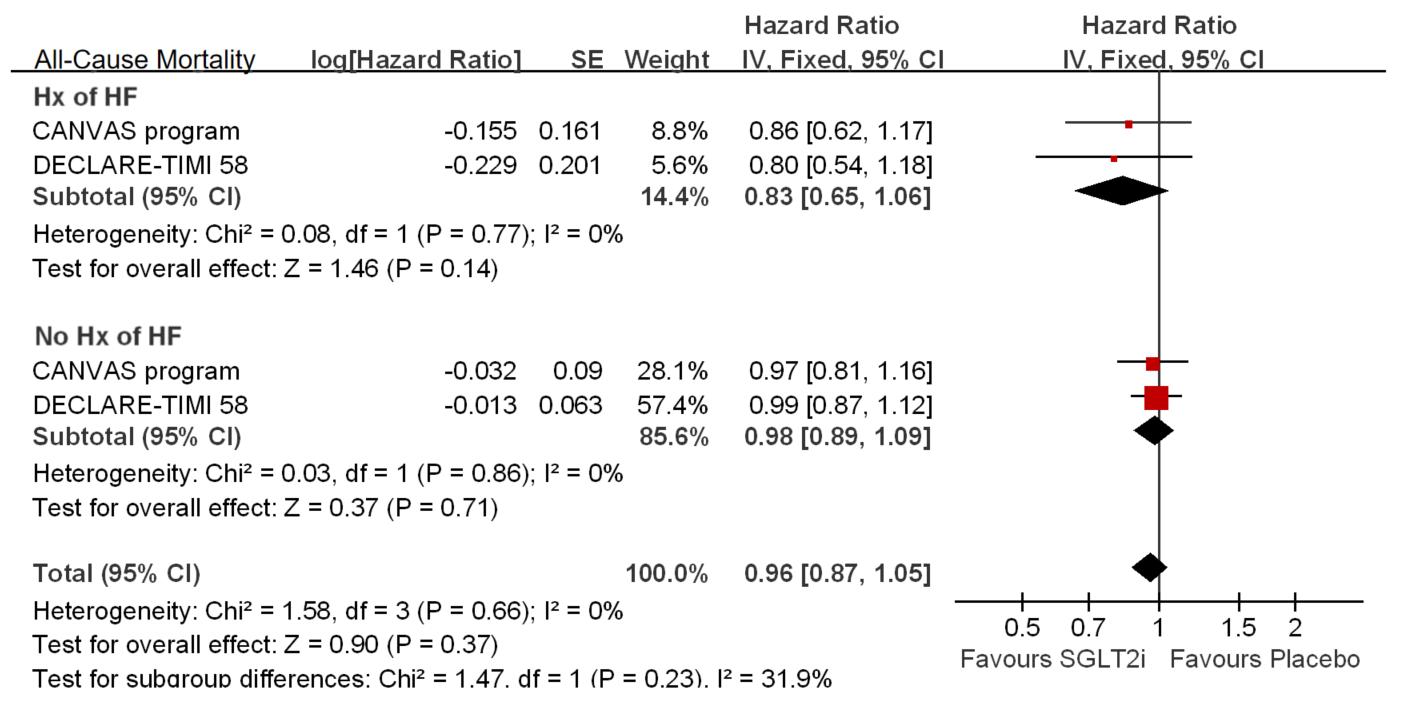

Supplement: Supplementary file 1 [file Data_Sheet_1.docx]
