# Supplementary material for: SGLT2 Inhibitors in Diabetic Patients With Cardiovascular Disease or at High Cardiovascular Risk: A Systematic Review and Meta-Analysis of Randomized Controlled Trials
Source: Front Cardiovasc Med. 2022 Apr 26;9:826684. doi: 10.3389/fcvm.2022.826684 (PMC9087280; doi:10.3389/fcvm.2022.826684)
Supplement: Supplementary file 2 [file Data_Sheet_2.docx]

**Identification of studies via databases and registers**

Records identified from PubMed, Embase, the Cochrane Library and clinicaltrial.gov (n = 2,444)

Additional records identified through other sources (n = 0)

**Identification**

Records after duplicates removed (n = 1,632)

Records excluded by titles and abstract (n = 1,468)

Full-text articles assessed for eligibility (n = 164)

Reports not retrieved (n = 146):

Duplicate articles (n = 26)

Not meet the meet inclusion criteria (n = 82)

Unclear and insufficient data (n = 38)

**Screening**

Studies included in qualitative synthesis (n = 18)

Studies included in meta-analysis (n = 4)

**Included**

*Consider, if feasible to do so, reporting the number of records identified from each database or register searched (rather than the total number across all databases/registers).

**If automation tools were used, indicate how many records were excluded by a human and how many were excluded by automation tools.

*From:*  Page MJ, McKenzie JE, Bossuyt PM, Boutron I, Hoffmann TC, Mulrow CD, et al. The PRISMA 2020 statement: an updated guideline for reporting systematic reviews. BMJ 2021;372:n71. doi: 10.1136/bmj.n71

For more information, visit: <http://www.prisma-statement.org/>
